# Enriched environments enhance the development of explicit memory in an incidental learning task

**DOI:** 10.1038/s41598-022-23226-5

**Published:** 2022-11-04

**Authors:** Carina Jaap, Marike C. Maack, Philipp Taesler, Frank Steinicke, Michael Rose

**Affiliations:** 1 NeuroImage Nord, Department for Systems Neuroscience, University Medical Center Hamburg Eppendorf, Martinistrasse 52, 20246 Hamburg, Germany; 2 Human-Computer Interaction, Department of Informatics, University of Hamburg, Vogt-Kölln-Str. 30, 22527 Hamburg, Germany

**Keywords:** Neuroscience, Psychology

## Abstract

Learning, rendered in an implicit (unconscious) or explicit (conscious) way, is a crucial part of our daily life. Different factors, like attention or motivation, influence the transformation from implicit to explicit memory. Via virtual reality a lively and engaging surrounding can be created, whereby motivational processes are assumed to be a vital part of the transition from implicit to explicit memory. In the present study, we tested the impact of an enriched virtual reality compared to two conventional, non-enriched 2D-computer-screen based tasks on implicit to explicit memory transformation, using an audio-visual sequential association task. We hypothesized, that the immersive nature of the VR surrounding enhances the transfer from implicit to explicit memory. Notably, the overall amount of learned sequence pairs were not significantly different between experimental groups, but the degree of awareness was affected by the different settings. However, we observed an increased level of explicitly remembered pairs within the VR group compared to two screen-based groups. This finding clearly demonstrates that a near-natural experimental setting affects the transformation process from implicit to explicit memory.

## Introduction

Learning is influenced by multiple factors, like attention, and motivation, and is rendered in an implicit (unconscious) or explicit (conscious) way^1,2^. Implicit memory is usually encoded incidentally so that regularities in the environment are extracted without the actual intention to learn them, and thereby influence our behavior without awareness. Evidence suggests, that implicitly acquired knowledge can become explicit, allowing us to extract and use regularities from the environment without having learned them consciously^3–5^. This memory transformation, from the implicit to the explicit domain, represents a crucial mechanism as learning such regularities and complex rules facilitates the development of higher cognitive functions such as reasoning and language^6^. This way, implicit learning forms the basis to adapt to a complex and changing world and guide the decision making in our daily life.

### The fluency hypothesis

Whether implicit information is transformed to become explicit and thereby conscious, depends on a multitude of factors^6–8^. One prominent theory to explain the transformation from incidental learning to explicit memory is based on the ongoing evaluation of predictions^6,9^. In particular, implicit perceptual associations result in predictions for upcoming events and these predictions are assumed to be used to monitor the outcome of consecutive processing. To study this effect, a sequential task is often implemented^6,10^. During the presentation of a predictable sequence of stimuli, a discrepancy between the predicted and the actual processing speed can be detected by the participants. Hence, this detection process can trigger attention towards the cause of this discrepancy and due to the consecutive search processes, explicit memory is generated^4,11^. This is in line with the Unexpected-Event theory^11^, which postulates that any metacognitive judgment, like unexpected fluency or accuracy, can be an unexpected event and trigger attributive processes. It can be assumed that this fluency of processing is enhanced in computer-screen-related tasks because, in more near-natural settings, the evaluation of stimuli and responses are slowed down by several factors. For example, in VR applications, the participants can explore the virtual environment to different as well as variable amounts of time. Additionally, the use of a controller as a response device slows down the response in contrast to classical response devices. These factors directly reduce the fluency of stimulus processing and should therefore affect the emergence of explicit memory.

Following this, our implemented 2D-computer-screen application probably creates more fluency of the responses, related to different factors, like smoother transition of trials compared to trials within the VR task design and therefore enable a rhythmical stimulus processing. However, the fluency in the computer-screen application is favored by the time needed for stimulus evaluation and response times due to different response devices. Following these assumptions, we hypothesized to find an enhanced emergence of explicit memory in a conventional 2D-computer-screen based environment in case that the fluency of stimulus processing is a crucial part of implicit to explicit memory formation. In the following we call this assumption, the *fluency hypothesis*. The contrary hypothesis, the *enriched environmental hypothesis*, can be formulated based on theories regarding learning within a more near-natural environment, assuming that rather realistic stimuli and context directly enhance explicit learning processes^12,13^.

### The enriched environmental hypothesis

The current cognitive research aspires to apply experimental designs in real-world settings (i.e. audio-visual processing while walking or performing daily activities^14,15^), as it was demonstrated that the context of learning (and retrieval) processes are highly relevant for their outcome^8,12,13^. Despite their advantages, these experimental settings are, however, extremely vulnerable to uncontrollable variables. To control external variables and provide a realistic setting, VR environments have become an attractive option. Previous applications demonstrated that these VR environments enable a sufficient, near-natural experience using interactive elements and multisensory stimulation, resulting in multiple levels of excitement and engagement^16,17^, which support an improved multi-sensory integration^18^. Moreover, VR facilitates a more salient processing of stimuli by dynamic engagement of the sensorimotor system, which provokes more naturalistic behavioral and physiological responses than abstract stimuli^19,20^. It was previously shown that learning in VR promotes better performance in an enriched environment^21,22^ and the literature demonstrated that these enriched stimuli are responsible for the motivational significance of stimuli^1,23,24^. This motivational effect can also enhance the detection of violated predictions due to an increased level of attention instead of performance fluency. Notably, compared to desktop PC settings, VR Head-Mounted Displays like the HTC Vive induce greater feelings of being present in the VR experimental surrounding, and higher motivation to interact with the environment^16,25^. So far, VR has already been broadly used in the research of explicit episodic and spatial learning (for a review see^22^). Here it has been shown that in contrast to basic computer setups, episodic memory performance is increased in VR settings^26,27^, which is most likely caused by the near-natural or more specifically immersive VR environments. Therefore, the benefit of using VR studies for explicit memory supports our assumption that VR can enhance the transformation from implicit to explicit memory***.*** It is an open question whether the emergence of explicit memory during incidental learning can also benefit from a more near-natural experimental setting or if this phenomenon is exclusive to task designs, in which the volunteers we instructed to memorize the stimuli. We expect, that the detection of unexpected events within ongoing prediction evaluations, needed for the transfer from implicit to explicit knowledge, benefits from the near-natural experimental application in VR reflected in increased explicit memory performance. This hypothesis, the *enriched environmental hypothesis*, is based on the existing evidence from the implicit learning domain and potential effects within a VR experience (i.e., rising motivational and attentional as well as engaging processes).

### The present study

In the present study, we employed a sequential-association task, which has been introduced in previous studies^6,28,29^. The task was used as a between-subjects design contrasting a conventional 2D-computer-based presentation with an enriched near-natural VR application. As the evaluation of stimuli and responses are probably slowed in the near-natural VR task, we tested our design within two different 2D-computer-screen groups, differing only in the number of trials, and one VR group. One 2D-computer-screen group practiced the identical amount of trials as presented in the VR condition, which operated as control group for the duration of VR condition and to exclude an influence of experimental length. Previous studies already demonstrated this smaller amount of trial in a 2D-computer-screen application lead to a partly generation of explicit memory in an incidental learning task^4,6^. Both 2D-computer-screen condition were control conditions for our fluency hypothesis.

The use of stimuli from different modalities (crossmodal) has been shown to be beneficial in learning paradigms because memory formation seems to benefit if encoded elements are derived from separate modalities, facilitating their integration (i.e., visual, and auditory^30–32^). The crossmodal stimuli implemented in the conventional 2D-computer-screen based task were modified from a previous study and consisted of simple tones and plain squares as visual stimuli^6^ (see Condition 2 for further details). The sequential regularities consisted of the presentation of alternating visual and auditory stimuli, building a fixed eight-digit sequence in 85% of the trials. Only in 15% of all trials this sequence was violated. The sequential task structure was unknown to the participants, and the content could only be learned incidentally.

Importantly, to assess the degree of explicit memory for the embedded sequences, an identical completion task^33^ and a free recall test were conducted at the end of all experimental conditions and always outside the VR. Thus, the results from these tasks can be directly compared between the different learning settings. Both post-tests were combined with a confidence rating^6^ to identify participants' explicit knowledge^5,8,34,35^. Previous applications revealed that participants with explicit memory express their knowledge with high confidence. The correct responses under high confidence are an indicator of explicit memory and, hence, will be used to differentiate them from implicit memory^36,37^. To test for probable differences in memory formation, based on the latter mentioned evaluations of the implemented stimuli in VR and the 2D screen conditions, a stimulus value rating was performed as a last step of the post-experimental assessment.

### Hypothesis

We hypothesized that both 2D-computer-screen groups perform similar, as it can be assumed that a *fluency* based unexpected event is detected in both variations of the experiments. Our modified version of the task for the VR contained images of different naturalistic landscapes and complex instrumental sounds. Furthermore, the enriched aspect of the VR was achieved by putting the participants directly into the naturalistic landscapes while performing the sequential task. Furthermore, we hypothesized that an enhanced explicit memory can be explained by two potential mechanisms. On the one hand, if *fluency* is an important factor in the transfer of implicit to explicit memory, we expected to find a boosted performance in explicit memory formation in the 2D-computer-screen application compared to the group performing the task in the VR condition. On the other hand, if the performance of explicit memory is greater within the VR condition, motivational and enriched environmental-based attention processes play a crucial role in naturalistic learning scenarios.

### Study aim

The aim of the present study was a direct comparison of both the *fluency* and *enriched environmental hypothesis* regarding the mechanism of incidental perceptual learning processes concerning the transfer from implicit to explicit memory. Therefore, we have contrasted a simple 2D-computer-screen experiment and a complex and enriched VR experiment. The influence of both hypotheses can be compared between the different experimental surroundings by assessing the degree of implicit and explicit memory after learning within two tasks that were identical for all experimental conditions.

## Material and methods

We tested a sequential association task within a VR environment as well as in two conventional 2D-computer-screen-based tasks. The conventional 2D-computer-screen based conditions will be called PC-short (260 trials) or condition 2 and PC-long (520 trials) or condition 3 in the following.

### Participants

102 (51 in cond. 1; 22 in cond. 2 and 29 in cond. 3) healthy participants with normal hearing and normal or corrected-to-normal vision took part in this study. Data of five participants had to be discarded as the participants did not complete the tutorial successfully (two in cond. 1, one in cond. 2 and two in cond. 3).

Data of 49 participants were part of the final analysis of the VR condition (29 Females, age M = 27.07 years). In the 2nd condition (PC-short), the datasets of 21 (11 Females, age M = 24.7 years) participants were included in the final analysis. In the 3rd condition (PC-long), the datasets of 27 (21 Females, age M = 27.6 years) participants were included in the final analysis (see Table 1). All experiment protocols were approved by the local Ethics Committee of the General Medical Council Hamburg (PV7022) and our methods were carried out in accordance with ethical guidelines and regulations. Before taking part in the experiments, all participants gave their written informed consent and were paid an expense allowance of 10 €/h.

**Table 1:** Overview of number of participants taking part in each condition before and after discarding relevant datasets.

Condition	N taking part in total	N of discarded datasets	N of datasets in the final analysis
VR	51	2	49
PC-short	22	1	21
PC-long	29	2	27

### Condition 1 (VR)

#### Apparatus

##### Inside the VR

The visual stimuli were presented in a virtual surrounding, built with the Unity 3.0 engine, via a head-mounted display i.e., HTC Vive. The responses were tracked with a HTC Vive controller. The acoustic stimuli were presented via headphones. The volume was adjusted by the participants to a comfortable level. The tutorial, as well as the main experiment, took place within the VR environment.

##### Outside the VR

After the main experiment, a completion task, a free recall, and a stimulus value rating were performed on a computer screen (23″, ~ 1 m distance to the participant) using a standard computer mouse. The tones were presented via two loudspeakers (HD 201, Sennheiser, Germany) one on each side of the screen. The volume of the acoustic stimuli was adjusted by the participants to a comfortable level during the before mentioned post-tests.

#### Stimuli

**Figure 1:**
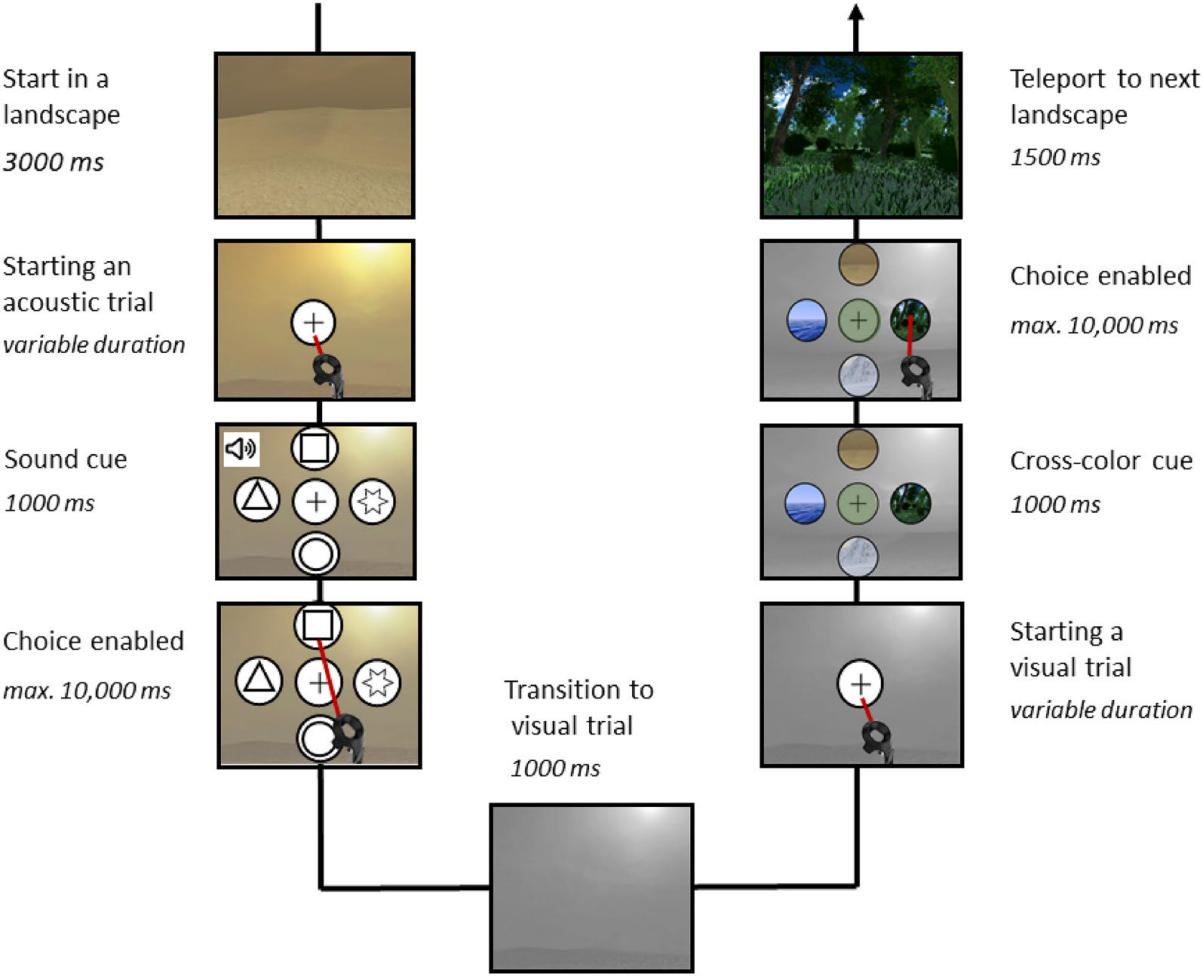
Timeline of a trial within the VR experiment (from upper left to the upper right). The participants were teleported to a virtual landscape where they had 3000 ms time for exploration. Afterwards, a white circle with a cross in the middle appeared in front of them, symbolizing the option to start a trial. The participants could autonomously start by pointing at the circle with the beam of their controller. Next, a sound was played for 1000 ms and four symbols occurred. The sound-symbol combinations were previously learned in the tutorial. Importantly, during the sound was played, participants could not respond. After the sound had ended, participants could choose one of the four symbols within 10,000 ms. After selecting the corresponding symbol, there was a transition from the acoustic to visual trial within 1000 ms during which the color of the current surrounding was desaturated. Afterwards, the visual trial started in the same way as the acoustic trial. Again, the participants could autonomously start the visual trial by pointing at the circle with the beam of their controller. Upon start, four visual stimuli symbolizing four landscapes appeared. Then the target circle in the middle changed from white to the dominant color of one of the four predefined landscapes within 1000 ms. During the color changing process, the participants could not give an answer. Next, participants had to select the icon representing the color of the target (i.e., if the target turned green, participants were expected to select the forest icon within 10,000 ms). After selecting a landscape icon, the participants were teleported to the next correct landscape, independent of their choice (i.e., if the participant chose the ocean landscape although the target was green, still the forest landscape was presented).

The visual stimuli in the VR consisted of four different landscapes (ocean, desert, ice, and forest). The participants were teleported into a landscape and were able to get a 360° view of the landscape. The landscapes were designed with the Unity engine. So, it was a fully immersive experience and not just a 360° image of the landscapes. The acoustic stimuli consisted of four complex instrumental tones, with a duration of 1000 ms each. Each sound was paired with a symbol (e.g. the piano sound was resembled as a star). By matching each sound with a specific symbol, the participants could match the played sound with the respective symbol within the experiment (see Fig. 1).

#### Experimental design and procedure

Before entering the main experiment, each participant was introduced to the stimuli and the task instructions for the main experiment during a tutorial. First, the participants had to learn the correct combination of tones and their corresponding symbol in a familiarization task. For the visual trials, the participants learned to match a color with one of the four landscape icons within a familiarization task. Each color was chosen in accordance to the dominant color of the corresponding landscape (desert = yellow). The tutorial ended when less than 2 errors over the last 10 trials were generated by the participants for each trial type. In the main experiment the participants were instructed to answer as fast and precise as possible in each visual and acoustic trial. The VR condition consisted of 260 teleports which is the equivalent of 520 (50% acoustic and 50% visual) trials. To avoid motion sickness, the participants were teleported slowly into the next scenery. The participants were offered several breaks during the VR experiment.

For a detailed overview of the timeline of trials within a teleport, representing a set of an acoustic and visual stimulus presentation in the main experiment, see Fig. 1.

As we were interested in the learning behavior within the VR, the acoustic and visual trial presentations were part of a sequence consisting of eight digits formed by pairs of eight different stimuli. Within the sequence, each visual stimulus (one of four landscapes) was paired with a specific sound (one of four musical instruments) e.g. visual 1: Desert with acoustic 1: Piano sound. Therefore, pairs or even the whole sequence could be learned in principle. The sequence was interrupted by deviants (15%) with a maximum of three in a row. The sequential regularity of the trial presentations was not introduced to the participants. To avoid motor learning, the order of the visual response option was randomized in each trial^33^.

#### Post-experimental assessment of knowledge and stimulus value rating outside the VR

**Figure 2:**
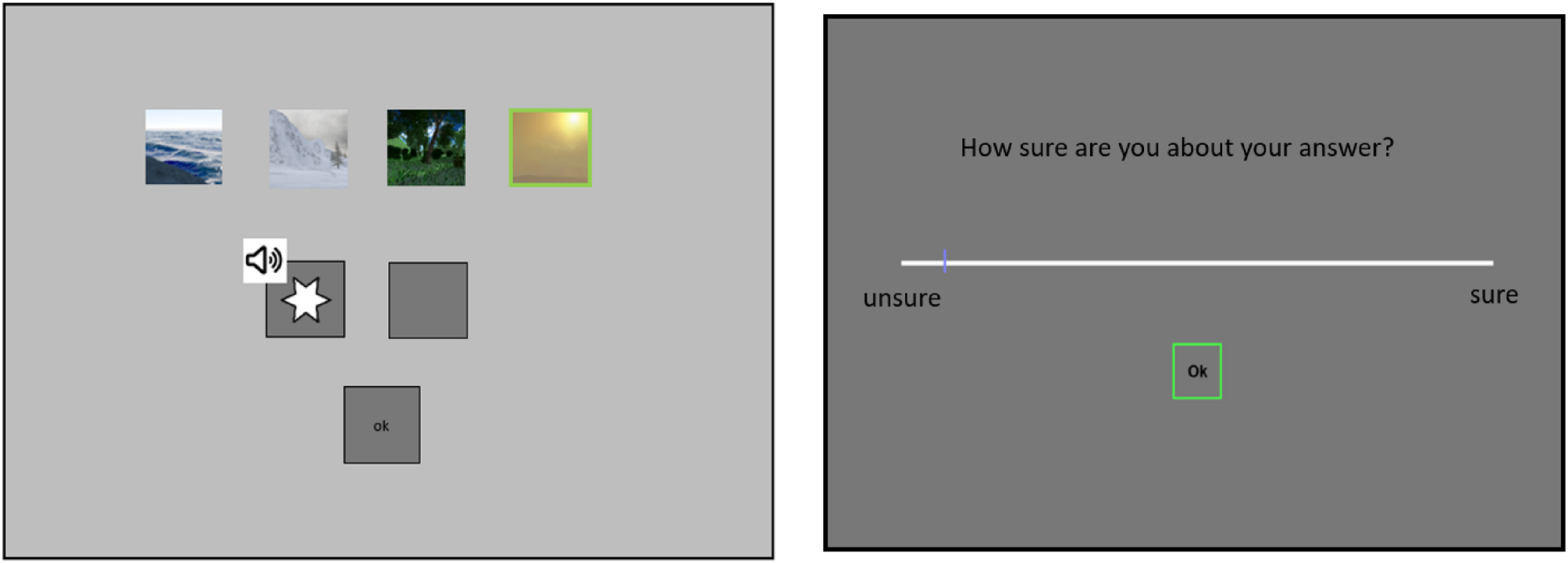
A completion task trial (left) and confidence rating (right) performed outside the VR. In the completion task, four stimuli were displayed above a given stimulus. The participants then had to match the correct visual or acoustic stimulus to the given one. Within the shown crossmodal completion task trial, the participants were asked to match the given acoustic stimulus with the surrounding they associate with it. The participants were asked to make a guess if necessary. After each trial in the completion task (as well as the free recall) the participants had to perform a confidence rating (left).

A completion task (see Fig. 2), a free recall, and a stimulus rating followed the main VR experiment. All post-experimental tasks were retrospectively performed outside the VR on a computer screen. In each trial, one of eight stimuli was given and had to be completed with a stimulus that matched the given stimulus (see Fig. 2). The trials were either crossmodal, with a given stimulus in one modality and four choices given from the opposite modality, or unimodal in which the given stimulus and the choices of answers were of the same modality. The completion task consisted of 64 trials with 50% crossmodal trials (25% visual and 25% acoustic matching). After each trial, the participants had to choose if they were sure or unsure about the given answer (see Fig. 2). With this rating, we later could separate the given answers into implicit (correct answer rated as unsure) and explicit (correct answer rated as sure) knowledge about the presented sequence.

The completion task was followed by a free recall. At this point, the participants were told that there was an order in which the stimuli were presented most of the time during the experiment. Within the free recall, the participants were asked to choose an order for the eight given stimuli. The chosen order should resemble a sequence the participants most likely were presented within the main experiment. The order for the chosen stimuli was not limited. After the participants logged their choice in, by clicking on an “ok” button, the participants were asked if they were sure or unsure about their chosen order.

At the end of the latter mentioned memory assessment tasks, the participants performed a stimulus value rating of the visual stimuli. We implemented a stimulus value rating to test for potential motivational significance of stimuli between the stimuli used in the visually enriched VR and the non-enriched screen based experiments, as this contextual influence can probably correlate with learning effects due to motivational and attentional factors^23^. Each visual stimulus was presented once and the participants could rate it on a continuous scale with “I dislike it” (negative rating) on the left, “Neutral” in the middle and “I like it” (positive rating) on the right side of the scale.

### Condition 2 (PC-short)

#### Apparatus

The visual stimuli were presented on a 23″ screen (SyncMaster P2370; Samsung). The distance between screen and participant was approximately 1 m. The tones were presented via two loudspeakers (HD 201, Sennheiser, Germany) one on each side of the screen. The volume was adjusted by the participants. For recording the answer of the participant, we used a standard keyboard and computer mouse. The psychtoolbox on Matlab was used to present the experiment.

The visual stimuli consisted of four colored and easily distinguishable squares. The color of each square was chosen analogue to a landscape within the VR condition (blue = ocean, green = forest, yellow = desert, white = icy landscape). Four simple tones (sine waves: 120 Hz, 286 Hz, 389 Hz and 527 Hz) with a duration of 1000 ms, were used as auditory stimuli^38^. Black circles of diameters ranging from 20 to 80% of the size of the visual stimulus were displayed as a visual response option for the acoustic stimuli. The circle size represented the frequency height e.g., the biggest circle represented the tone with the lowest frequency.

#### Experimental design and procedure

The participants were instructed to respond as quickly and correctly as possible to the target stimulus which was presented in the centre of the screen for the visual stimuli or as a tone to which the participants had to match one of four circles. The participants underwent a training before entering the main experiment (see condition 1). The response options were displayed above the target. The last visual target was still present during an acoustic trial to keep it analogue to the setting in the VR experiment in which the participants remained in a landscape during the acoustic trial (see Fig. 1). After the participants gave their response, the trial ended. Answers had to be given within 2500 ms per trial.

Responses had to be made with the index and middle finger of both hands on a regular keyboard. The enabled keys were “y”, “x”, “, ; “ ,and “. : “.

Condition 2 or PC-short consisted of 260 trials. The trials were part of a sequence consisting of eight stimuli, four in each condition starting with a visual stimulus (Sequence: V1 (e.g. blue square) A1 (e.g. 286 Hz), V2A2 V3A3 V4A4; for more details, see Condition 1).

#### Post-experimental assessment of knowledge and stimulus value rating

A completion task, a free recall and a stimulus rating followed the main experiment. All post-experimental tasks were performed on a computer screen and were identical, with an exception for the used visual and acoustic stimuli, to the tasks performed by the experimental group 1 (see cond. 1, VR).

### Condition 3 (PC-long)

#### Apparatus

See cond. 2.

#### Stimuli

See cond. 2.

#### Experimental design and procedure

The procedure was the same as in condition 2 except that condition 3 consisted of 520 instead of 260 trials. See cond. 2 for further details.

#### Post-experimental assessment of knowledge and stimulus value rating

See cond. 1 and cond. 2.

### Behavioral data analysis

The important parameters, which can be compared between all experimental conditions, are the amount of memory expressed as implicit or explicit memory. These parameters can be taken from the completion task, as well as the free recall. The latter tasks were identical for all three conditions, except for a change in both visual and acoustic stimuli in the VR condition. All correct answers were taken into account for the analysis of task performance in the completion task, as well as the free recall. Within the main experimental conditions, specific acoustic and visual stimuli formed pairs within a sequence in 85% of the trials. We counted an answer as correct if the participant was able to match a given stimulus with a stimulus of the other modality that was either the following or the previous stimulus within the sequence. As the last visual stimulus was present, when the acoustic stimulus was presented and vice versa, we assumed, that not only forward but also backward learning within the sequence was feasible. Therefore, we accepted an answer in both directions. The amount of implicit memory was calculated as the percentage of “unsure”, correct, answers from the sum of all possible answers per participant. The amount of explicit memory was calculated as the percentage of “sure”, correct, answers from the sum of all possible trials per participant. This method was used for the assessment of the type of acquired knowledge (implicit; explicit) in the different conditions in both the completion task as well as in the free recall.

To test for differences in the amount as well as quality of gained knowledge, we performed an ANOVA with the factors Condition (VR; PC-short; PC-long) and Learning-Type (implicit; explicit) with performance in each learning type as the dependent variable within the completion task as well as the free recall. Furthermore, we tested for probable differences in the quality of gained knowledge between the two conventional 2D-screen-based tasks and performed an ANOVA with the factors PC-Conditions (PC-short; PC-long) and Learning-Type (implicit; explicit) with performance in each learning type as the dependent variable within the completion task as well as the free recall. For the analysis of the stimulus value rating, all given answers were taken into account. We controlled for potential outliers, i.e. participants selecting only “sure” and while showing constant errors, before we went on with the further analysis. For each rating, where a value between 0 (unpleasant) and 1 (pleasant) was possible, the absolute distance to the neutral rating (0.5) was calculated. A mean over these adjusted stimulus ratings of the four visual stimuli was calculated for each participant in each condition and used for the further analysis. To test for a probable impact of the enriched stimuli, used in the VR, on the stimulus value rating, we performed a two-sided two-sample t-test over stimulus ratings in VR versus PC (PC-short & PC-long merged) conditions. Furthermore, we tested for probable correlations of stimulus value ratings on implicitly and explicitly gained knowledge in each group. The statistical analysis was performed in R (4.0.5) and Matlab (2020b).

## Results

### Completion task

Notably, we found no difference in the amounts of overall learned sequence pairs between conditions (see Table 2), but the degree of developed explicit memory was affected by the different settings in the three conditions.

**Table 2:** Completion task performance over learning types in each condition.

Type	Conditions					
	VR [%]	SEM [%]	PC-short [%]	SEM [%]	PC-long [%]	SEM [%]
Implicit	19.2	3.15	45.8	5.44	52	2.5
Explicit	36.7	4.2	14.4	4.4	4.62	1.5
Total learned	56	3.12	60.3	3.52	56.6	2.4

Mean and SEM in percent for the performance between and within conditions (VR = 49, PC-short = 21, PC-long = 27) for Learning-Type (implicit; explicit) and over all learned stimulus pairs.

**Figure 3:**
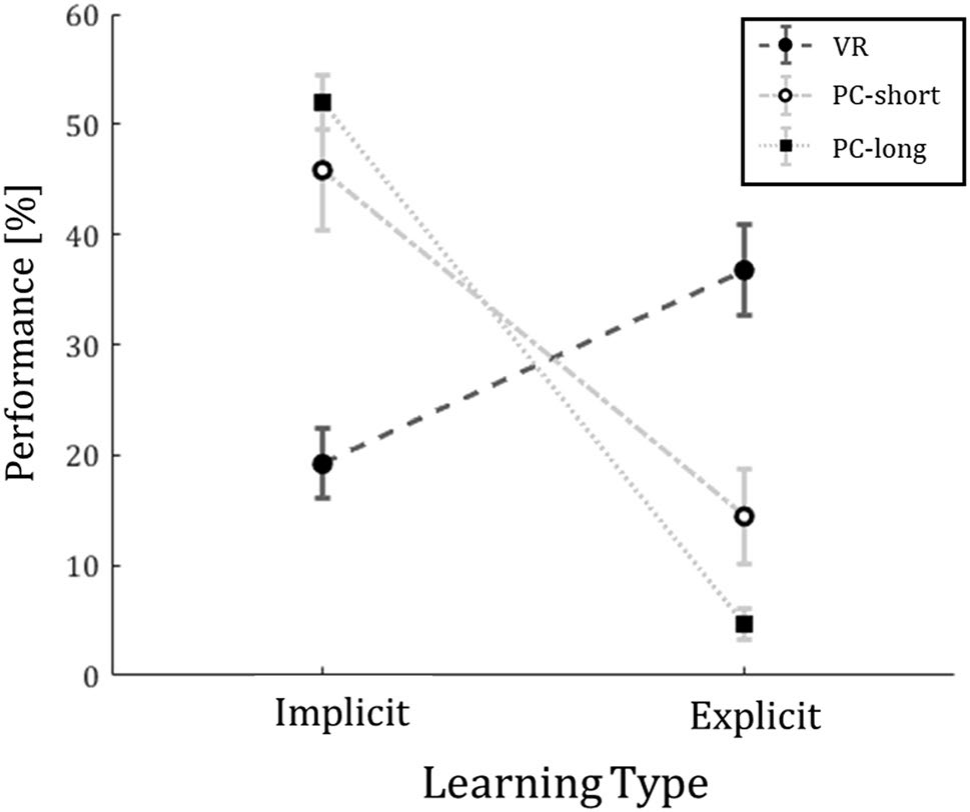
Completion task performance of learned pairs within the given sequence divided by Learning-Type (implicit; explicit) and Condition (VR, PC-short, PC-long). The light grey, dotted lines represent the performance of participants of PC-short (N = 21) and PC-long (N = 27) and the dark grey, dashed line represents the performance of VR (N = 49) participants in the completion task. The performance is divided into implicitly learned sequence pairs (left) and explicitly learned sequence pairs (right). The mean of the performance is visualized as black circle (VR), hollow circle (PC-short) and black square (PC-long). The error bars represent the SEM.

The interaction effect of Conditions and Learning-Type (F_(2188)_ = 27.3, *p* < 0.0001; see Fig. 3) revealed more explicit memory in the VR group as compared to both PC versions. Overall, volunteers acquired more implicit than explicit memory (main effect of Learning-Type: F_(1188)_ = 23.1, *p* < 0.0001).

### Free recall

**Figure 4:**
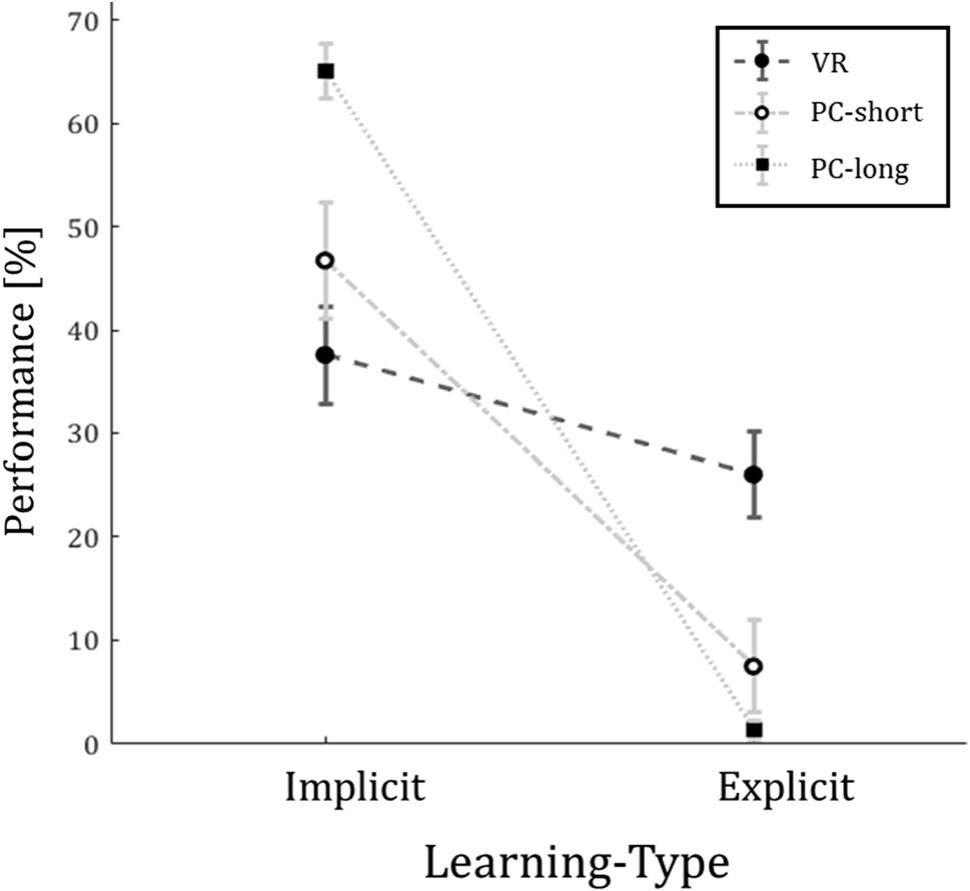
Free recall performance of learned pairs within the given sequence divided by Learning-Type (implicit; explicit) and Condition (VR, PC-short, PC-long). The light grey, dotted lines represent the performance of participants of PC-short (N = 21) and PC-long (N = 27) and the dark grey, dashed line represents the performance of VR (N = 49) participants in the free recall. The performance is divided into implicitly learned sequence pairs (left) and explicitly learned sequence pairs (right). The mean of the performance is visualized as black circle (VR), hollow circle (PC-short) and black square (PC-long). The error bars represent the SEM.

Notably, we found no difference in the amounts of overall learned sequence pairs between conditions (see Fig. 4), but the degree of awareness was affected by the different settings between conditions.

In the free recall, the VR experimental group had a mean performance of 37.57% for implicit and 26.03% (SEM = 3.61%) for explicit memory. In contrast, the conventional, 2D screen based experimental groups resulted in an average performance of 46.73% implicit and 7.44% explicit memory (PC-short; SEM = 5.12%) and 65.06% implicit and 1.27% explicit memory (PC-long; SEM = 4.87%).

Overall, the volunteers acquired more implicit than explicit memory (main effect of Learning-Type: F_(1188)_ = 55.9, *p* < 0.0001). The interaction effect of Conditions and Learning-Types (F_(2188)_ = 10.9, *p* < 0.0001; see Fig. 4) revealed more explicit memory in the VR group as compared to both PC groups.

### Gained knowledge in the short versus long version of the 2D-computer-screen based conditions

We tested for probable differences in the mean of gained explicit and implicit knowledge between a short and a long version of the 2D-computer-screen based sequential-association-task. Notably, we found no difference in the amounts of overall learned sequence pairs between the 2D-computer-screen based conditions. Overall, volunteers acquired more implicit than explicit memory in both the completion task (main effect of Learning-Type: F_(1,92)_ = 82.94, *p* < 0.0001; see Fig. 3) as well as the free recall (main effect of Learning-Type: F_(1,92)_ = 265.2, *p* < 0.0001; see Fig. 4). An interaction effect for the influence of Conditions PC-short versus PC-long on Learning-Type was significant for both completion task (F_(1,92)_ = 12.44, *p* < 0.001) and free recall (F_(1,92)_ = 4.770, *p* < 0.0315) (see Figs. 3 and 4). However, this effect is related to less explicit memory in the PC versions.

### Stimulus value rating

We tested for probable differences in the mean of stimulus value ratings between an enriched visual environment in the VR task and non-enriched visual stimuli in both PC tasks.

**Figure 5:**
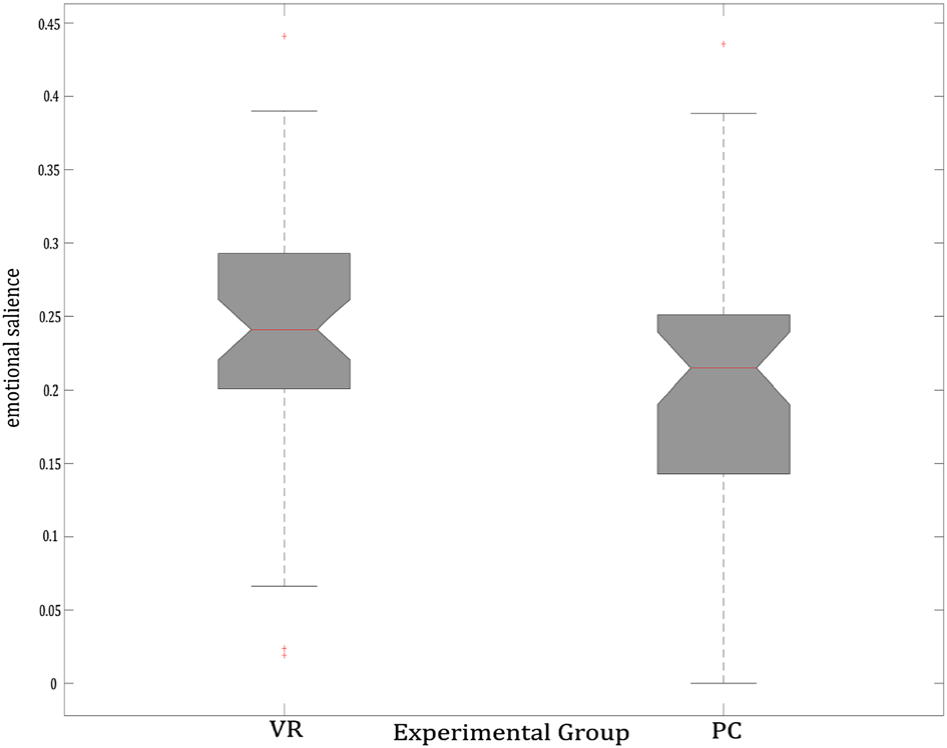
The stimulus value rating for the VR condition and the two merged 2D-computer-screen conditions. The boxplot shows for the VR condition a median of 0.24 (25-percentiles of 0.2 and 75-percentiles of 0.29) and 0.21 (25-percentiles of 0.14 and 75-percentiles of 0.25) for the computer-screen conditions.

We could not find a statistically significant difference in stimulus value ratings (t_(95)_ = 1.82, *p* = 0.071) between conditions of VR (Mean = 0.24; SEM = 0.012) compared to both conventional 2D-computer-screen based conditions taken together (Mean = 0.21; SEM = 0.015; see Fig. 5). Furthermore, we tested for associations of stimulus values and the amount of implicit and explicit knowledge separately for each task. We found no correlation of the stimulus value ratings and the performance of implicit memory, as well as no correlation between the stimulus value ratings and the performance of explicit memory (all *p* > 0.05).

We additionally calculated an ANCOVA for both post-experimental assessments thereby the stimulus value rating functions as the covariate to test whether the interaction effect is affected by the stimulus value. We gained comparable significant effects to the previous performed ANOVA. The ANCOVA for the completion task resulted in an interaction effect between Condition and Learning-Type (F (2188) = 2.82, *p* < 0.0001) and a main effect for Learning-Type: F (1188) = 12.21, *p* < 0.0001). The ANCOVA for the free recall resulted in an interaction effect between Condition and Learning-Type (F (2188) = 31.79, *p* < 0.0001) and a main effect for Learning-Type: F (1188) = 73.45, *p* < 0.0001).

## Discussion

Although incidental learning is a fundamental process occurring in everyday life, its underlying mechanism is commonly investigated in artificial laboratory environments. In particular, the potential benefits for the generation of explicit memory during learning in naturalistic contexts remain elusive. In this study, we aimed on closing this gap by implementing a near-natural sequential association task in an enriched environment presented in VR. We compared the generated knowledge in the enriched task environment with the learning behavior we got when participants perform a sequential association task in a classical and non-enriched environment. Our results revealed that participants successfully acquired a comparable amount of memory in both enriched and non-enriched learning environments.

### Enhanced explicit memory formation in an enriched task design

Although the amount of learned items was similar across all applications, two memory tests (completion and free recall task) revealed that the extent of later expressed explicit memory was enhanced in the enriched environment compared to the conventional non-enriched environment. These results support the view that an enriched setting is an essential factor that can explain an increased explicit memory in the VR application of the incidental sequential association task. Therefore, the *enriched environmental hypothesis* is supported by our results. We could not find a significant difference in the visual stimulus value rating used in the VR condition compared to the conventional 2D-computer-screen based applications, indicating that the stimulus alone outside the VR cannot explain the difference in the explicit memory generation. Therefore, the enhanced development of explicit memory can be related to various advantages of the VR application^19,21,22,39^. One possible factor includes that VR applications facilitate the transition of information by its general characteristics in mobility and natural movements like the upright body position, and not only arms but especially head movement freedom. That way, VR applications might reflect a motivating environment with the potential to increase the feeling of an embodiment, like being physically present in the scenario, by which encoding efficiency and the transition into explicit memory are increased^25,40^. Closely related, evidence from Smith & Mulligan (2021)^25^ includes the concept of immersion, which represents the degree of natural features in the VR portrayed by the virtual environment's sensory and interactive properties (see^41,42^). Previous research showed that immersion strongly correlates with participants' attentional engagement level^43,44^. Hence, the VR application not only provides a less vulnerable setting to attention decreases (i.e., due to mind wandering), it might even enhance attentional processes^16,25,45^. These advantages are a potential explanation for the enhanced explicit memory in the VR condition and constitute to the higher enriched environmental aspects that are increased in more real-life settings. Besides the environment posing an essential factor, near-natural perception is also increased when stimulus material is enriched by context-related components, enabling superior memory formation^46–48^. These factors of the *enriched environmental hypothesis* have a clear impact on the transition from implicit to explicit memory.

### The role of motivation in learning and why an enriched environment facilitates explicit memory formation by boosting motivational and attentional processes

In neuroscience, recent evidence has specifically connected the ventral striatum as a key player in the transition mechanism of implicit to explicit memory formation in incidental learning. The ventral striatum is mainly associated to motivational and reward processes^49^ releasing dopamine in rewarding situations, consequently enhancing the generation of explicit memory. In a study by Clos et al. (2018)^10^, which also included a sequential task, the dopaminergic level was pharmacologically modulated in human adults. It was shown that an increase in dopamine was directly connected to an increased transfer of information. This increase in information transfer was linked to the enhanced formation of explicit memory in an incidental sequential task. This way, successful predictions during task processing reflect an achievement within our neural system rewards. These rewarding processes encourage insight into hidden regularities and the emergence of explicit memory. The role of the dopaminergic system in the transfer from implicit to explicit memory may be related to the increased explicit memory rate in the present study as a consequence of the more rewarding and motivating experimental setting in an enriched task environment like we introduced to the participants in the VR surrounding.

### The fluency hypothesis versus the enriched environmental hypothesis

To control for the feeling of task fluency (see the introduction for further details), we implemented two simple conventional 2D-computer-screen based versions, which differed in experiment length. The increased amount of implicit memory in the 2D-computer-screen condition indicated that the content is still learned, but is transferred to explicit memory only to a reduced degree. By directly comparing the effect of the *fluency* factor and the *enriched environmental* factor in the present experiment, the impact of the motivational and enriched experimental environment seems to have a larger effect on the emergence of explicit memory.

### Conclusion

To summarize, the increased transition from implicit to explicit memory during incidental learning in the VR setting is related to an interaction of enhanced task processing, rewarding processes and attentional as well as motivational factors. These observations in the VR application have strong therapeutic implications for the rehabilitation of patients, who suffer from learning and memory impairments according to neurological diseases. We show that the transition from implicit to explicit memory is considerably influenced by the enriched environment, which includes context-enriched stimuli. In this work, we have obtained compelling evidence that the transition from implicit to explicit memory is primarily influenced by environment-engaging processes like attention, motivation, and presence.

### Limitations of this study

Within this study, we gained evidence that the environment used for studying incidental learning in humans influences the outcome of the type of generated knowledge to a great extent. As this study is one of the first attempts in elucidating the complex processes such an enriched and near-natural study design, here presented in VR, can have on incidental learning, we cannot clearly state which of the factors mentioned within our discussion plays a key role that led to the enhanced transfer from implicit to explicit memory formation. Yet, the findings emphasize the relevance for investigating learning and memory processes in more near-natural scenarios.

## Data Availability

The datasets generated and/or analyzed during this study are available on request from the corresponding author.

## Abbreviations

ITI: Intertrial interval
SEM: Standard error of the mean
VR: Virtual reality
